# Phenotypic and Genotypic Co-receptor Tropism Testing in HIV-1 Epidemic Region of Tanzania Where Multiple Non-B Subtypes Co-circulate

**DOI:** 10.3389/fmicb.2021.703041

**Published:** 2021-07-07

**Authors:** George P. Judicate, Godfrey Barabona, Doreen Kamori, Macdonald Mahiti, Toong Seng Tan, Seiya Ozono, Amina Shaban Mgunya, Takeo Kuwata, Shuzo Matsushita, Bruno Sunguya, Eligius Lyamuya, Kenzo Tokunaga, Takamasa Ueno

**Affiliations:** ^1^Joint Research Center for Human Retrovirus Infection, Kumamoto University, Kumamoto, Japan; ^2^Muhimbili University of Health and Allied Sciences, Dar es Salaam, Tanzania; ^3^Department of Pathology, National Institute of Infectious Diseases, Tokyo, Japan; ^4^Muhimbili National Hospital, Dar es Salaam, Tanzania

**Keywords:** HIV-1, envelope, entry, co-receptor tropism, non-B subtypes, inter-subtype recombinant form

## Abstract

HIV human immunodeficiency virus type I (HIV-1) entry inhibitor potency is dependent on viral co-receptor tropisms and thereby tropism determination is clinically important. However, phenotypic tropisms of HIV-1 non-B subtypes have been poorly investigated and the genotypic prediction algorithms remain insufficiently validated. To clarify this issue, we recruited 52 treatment-naïve, HIV-1-infected patients in Tanzania, where multiple HIV-1 non-B subtypes co-circulate. Sequence analysis of 93 infectious envelope clones isolated from their plasma viral RNA revealed the co-circulation of subtypes A1, C, D, and inter-subtype recombinant forms (isRFs). Phenotypic tropism assays revealed that lentivirus reporters pseudotyped with 75 (80.6%) and 5 (5.4%) envelope clones could establish infection toward U87.CD4 cells expressing CCR5 (R5) and CXCR4 (X4), respectively; whereas the remaining 13 (14%) clones could infect both cells. Genotypic analyses by widely used algorithms including V3 net charge, Geno2pheno, WebPSSM, and PhenoSeq showed that almost all phenotypic X4-tropic clones and only 15 of 75 phenotypic R5-tropic clones were concordantly predicted. However, the remaining 60 phenotypic R5-tropic clones were discordantly predicted by at least one algorithm. In particular, 2 phenotypic R5-tropic clones were discordantly predicted by all algorithms tested. Taken together, the results demonstrate the limitation of currently available genotypic algorithms for predicting co-receptor inference among co-circulating multiple non-B subtypes and emerging isRFs. Also, the phenotypic tropism dataset presented here could be valuable for retraining of the widely used genotypic prediction algorithms to enhance their performance.

## Introduction

HIV-1 infects host cells in a systematic multi-step entry process, initiated by HIV-1 envelope surface glycoprotein (gp120) attachment to the host CD4 receptor then to a co-receptor, either CCR5 (R5) or CXCR4 (X4), followed by membrane fusion and the release of virus particles into the host cell cytoplasm. HIV-1 strains show differential preference of attachment to co-receptor that led to their classification into R5-tropic, X4-tropic and dual R5X4-tropic ones (Moser, 1997; Wilen et al., 2012; Chen, 2020). The co-receptor usage dynamics in HIV-1-infected patients was initially thought, based on studies using subtype B infections, that the R5-tropic virus dominates during the early phase of the disease and that almost half of these patients eventually harbor X4-tropic ones in later disease stages (Ndung’u et al., 2006). However, it is becoming evident that the co-receptor usage dynamics is highly dependent on the infecting subtypes (Schuitemaker et al., 2010). Specifically, patients with subtype D infection tend to harbor X4/R5X4 tropic viruses even in the early phase of their disease (Kaleebu et al., 2007); whereas those with subtype C rarely harbor X4/R5X4 tropic viruses at any disease stage (Ndung’u et al., 2006; Huang et al., 2007; Lin et al., 2011, 2012). In another aspect, antiviral activity of the entry inhibitors are generally dependent on viral co-receptor tropism (Donzella et al., 1998; Baba et al., 1999; Lalezari et al., 2003; Dorr et al., 2005; Lobritz et al., 2010); e.g., Maraviroc and AMD3100 inhibit the entry of R5-tropic and X4-tropic viruses, respectively (Donzella et al., 1998; Dorr et al., 2005). Therefore, testing for viral tropisms is important for not only understanding viral characteristics but also useful in the clinical setting, where Maraviroc is used as a salvage therapy.

Co-receptor usage of a given viral envelope lineage can be definitively determined by a phenotypic assay, which requires specific amplification of the DNA of a full-length envelope gene, preparation of pseudovirus harboring those envelope glycoproteins, and infectivity assessment using CD4^+^ target cells expressing either R5 or X4 (Whitcomb et al., 2007; Lin et al., 2010; Raymond et al., 2010). This process is cost ineffective, highly laborious, and time consuming; and thereby it is not suitable when large numbers of samples are being tested for co-receptor usage. Instead, several genotypic prediction algorithms have been developed to infer the co-receptor usage based on amino acid sequence information on the hypervariable region 3 (V3) sequence of the envelope gene. However, many of the widely used genotypic prediction algorithms including Geno2pheno, WebPSSM, and PhenoSeq were developed mainly from subtype B information and show relatively poor performance when applied to non-B subtypes. Although algorithms optimized for non-B subtypes including SCOTCH (Löchel et al., 2018), CoRSeqV3-C (Cashin et al., 2013), and THETA (DImeglio et al., 2020) have been attempted, the co-receptor tropism prediction of co-circulating multiple subtypes and emerging inter-subtype recombinant forms (isRFs) remains challenging (Taylor et al., 2008). Thus, it could be of value to document the phenotypic co-receptor tropisms of a diverse panel of HIV-1 variants across multiple non-B subtypes and emerging isRFs that co-circulate in a given geographical region. In this study, we analyzed phenotypic tropism and genotypic prediction of viral co-receptor tropism in a diverse panel of 93 envelope clones isolated from 52 treatment-naïve, HIV-1-infected patients from Tanzania, where multiple non-B subtypes and various isRFs are known to co-circulate.

## Materials and Methods

### Study Participants

Fifty-two self-reported treatment-naïve patients were recruited between June 2017 and June 2018 after their initial diagnosis at the Care and Treatment Clinic (CTC) at Muhimbili National Hospital or at Mnazi Mmoja Hospital, Dar es salaam, Tanzania. Clinical and geographical information about the study participants is given in Table 1. The study protocol was approved by the Senate Research and Publications Committee of Muhimbili University of Health and Allied Sciences (Ref.No.2016-12-07/AEC/Vol.XI/325) and the National Health Research Ethics Committee of the National Institute of Medical Research, Tanzania (NIMR/HQ/R.8a/Vol.IX/2373). Written informed consent was obtained from all study participants.

**TABLE 1:** Summary of demographic, clinical, and viral characteristics.

	Total	Subtypes				*P* value
		A1	C	D	isRF	
**Demographic data**						
Patient, % (*N*)	100 (52)	28.8 (15)	23.1 (12)	13.5 (7)	34.6 (18)	
Age, median years (IQR)	38 (32–45)	39 (34–46)	31 (24–42)	39 (35–39)	40.5 (32.75–45.50)	0.26^b^
Female, % (*N*)	78.8 (41)	80.0 (12)	66.7 (8)	85.7 (6)	83.3 (15)	0.0054^a^
**Clinical data**						
Viral load, log copies/ml	5.13 (4.65–5.66)	5.13 (4.70–5.76)	5.08 (4.39–5.43)	5.06	5.66	0.88^b^
Median (IQR)	*5 missing*	*2 missing*	*1 missing*	(4.03–5.48)	(4.82–6.02)	
CD4^+^ count, cells/mm^3^	307.5 (135.5–477)	387 (235–538.5)	95 (40–240)	392	243 (123–364.5)	0.23^b^
Median (IQR)	*34 missing*	*9 missing*	*9 missing*	*6 missing*	*10 missing*	
Recently infected, % (*N*)	17.3 (9)	6.7 (1)	16.7 (2)	42.9 (3)	16.7 (3)	<0.001^*a*^

*^*a*^Chi-Square test. ^*b*^Kruskal–Wallis test.*

### Analysis for Duration of Infection

Duration of HIV-1 infection was assessed by performing a limiting antigen avidity enzyme immunoassay incorporating a recombinant protein containing the major variants of the gp41 immunodominant regions among the HIV-1 group M viruses (Asanté HIV-1 Rapid Recency Assay, Sedia BioScience Corporation, United States). Plasma samples that had been separated by centrifugation from whole blood collected from each study participant into EDTA-coated tubes were used for this assay. Recent infection inferred to be less than 180 days was estimated according to the manufacturer’s protocol.

### Cloning and Sequencing of Envelope Genes

Plasma viral RNA was extracted by using a QIAMP Viral RNA Mini Kit (QIAGEN), and the DNA encoding the entire envelope region was amplified with reverse transcriptase-coupled PCR as described before (Barabona et al., 2019). The primers and the HIV-1 genome coordinates (based on HXB2 strain) were as follow: (forward) 5′-GGTCAGGGAGTCTCCATAGAATGGAGG-3′ (5,284–5,311) and (reverse) 5′-GCACTCAAGGCAAGC TTTATTGAGGCTTA-3′ (9,144–9,171) for the 1st round reaction followed by nested PCR with primers 5′-TTAGGCATCTCCTATGGCAGGAAGAAGCGG-3′ (forward) (5,957–5,987) and 5′-GGGAGGGAGAGGGGCTTTGACCACT TGCCACCC-3′ (reverse) (8,816–8,799) or another set of the primer pairs, 5′-CTTGGTACCGAGCTCGTGGAAGCCATAAT AAGAATTCTGCAACAA-3′ (forward) (5,727–5,763) and 5′-GGGAGGGAGAGGGGCGATCTACAGCTGCCTTGTAAGTC ATTG-3′ (reverse) (9,032–9,055) were used for the second reaction, as needed. The resultant amplicons were cloned into an expression plasmid by use of a Gibson Assembly homologous recombination system (NEB). The median of four envelope clones per patient were pseudotyped and tested for infectivity toward TZM-bl cells. The clones showing infectious potential (see below) were selected, sequenced by the Sanger method, as previously described (Montefiori, 2009; Mwimanzi et al., 2013; Barabona et al., 2019), and used for further analyses. Viral subtypes were analyzed by using REGA HIV-1 subtyping tool v.3.0.^1^ Maximum likelihood phylogenetic tree and pairwise genetic distances for entire envelope sequences between clones from the same patient were calculated by MEGA (6) using the Tamura-Nei model, with standard error estimates obtained by performing the Bootstraps procedure with 1,000 replicates and incorporated nucleotide substitution including transition and transversions, as previously described (Bello et al., 2007).

### Construction of Proviral DNA

To generate pSG3.1 (NIH AIDS Reagent Program, United States)-based HIV-1 proviral DNA carrying a luciferase reporter gene and HiBiT tag, the *Nco*I/*Nco*I fragment of pSG3.1 harboring integrase-vpr genes was subcloned into *Nco*I-digested pcDNA3.1, and the *Bam*HI site was introduced by QuikChange mutagenesis (Stratagene) into the C-terminal end of the integrase gene of this subclone using the following specific oligonucleotides (restriction enzyme sites underlined); 5′-GGA TGA GGA TCC GAA CAT GGA TAA G-3′ and its antisense. The resultant plasmid was digested with *Bam*HI, and used for the insertion of an oligonucleotide linker corresponding to HiBiT-tag (5′-GAT CTT GTC AGT GGC TGG AGG CTC TTC AAG AAG ATT AGC TAG-3′ and 5′-GAT CCT AGC TAA TCT TCT TGA AGA GCC TCC AGC CAC TGA CAA-3′). The *Nco*I/*Nco*I fragment of the resultant vector was cloned back into pSG3.1, in which the *Not*I site was introduced by QuikChange mutagenesis into the N-terminal end of the nef gene using the following specific oligonucleotides; 5′-GCT TTT GCT ATA AGC GGC CGC GCA AGT GGT CAA AAC-3′ and its antisense. The resultant plasmid was digested with *Not*I, and used for the insertion of the *Not*I-digested luciferase (Luc2) fragment amplified using the following specific oligonucleotides; 5′-CAT TAG CGG CCG CCA TGG AAG ATG CCA AAA ACA T-3′/5′-CAT TAG CGG CCG CTT ACA CGG CGA TCT TGC CGC-3′, and the generated plasmid was designated pSG3_Δ__ENV__Δ__Nef_-Luc2-IN/HiBit.

### Phenotypic Co-receptor Tropism Assay and Entry Inhibitor Sensitivity

The reporter lentivirus pSG3_Δ__ENV__Δ__Nef_-Luc2-IN/HiBit was pseudotyped with a series of patient-derived envelope clones, by co-transfection of HEK-293T cells (7 × 10^5^ per well) as previously described (Wei et al., 2002; Ding et al., 2017; Ozono et al., 2020). Forty-eight hour later, the supernatants containing pseudoviruses were harvested and stored at –80°C until use. The envelopes of NL43 and JRFL were used as controls for X4 and R5 entry, respectively. The amount of the pseudovirus preparation was quantified as follows; the pseudovirus stock with known levels of p24 antigen derived from the pSG3_Δ__ENV__Δ__Nef_ reporter was serially diluted for a standard curve. Either the standards or the samples of interest (25 μl) and LgBiT protein (1:100)/HiBiT Lytic Substrate (1:50) in Nano-Glo HiBiT Lytic Buffer (25 μl) (Nano-Glo HiBiT Lytic Detection System; Promega) were mixed and incubated for 10 min at room temperature according to the modified manufacturer’s instructions. Luciferase activity was determined with a luminometer. The phenotypic co-receptor usage was then analyzed as previously described (Huang et al., 2007). In brief, U87.CD4.CCR5 and U87.CD4.CXCR4 cells (1 × 10^4^ per well) (NIH AIDS Reagent Program, United States) were exposed to the pseudoviruses (1 ng) for 48 h in medium supplemented with 10 μg/ml of diethyl amino ethanol (DEAE). The cells were lysed for the measurement of firefly luciferase activity by using a ONE-Glo Luciferase Assay system (Promega, United States). Relative luminescence unit (RLU) was determined by subtracting the luminescence values in the absence of target cells in the reaction.

Sensitivity of the pseudoviruses toward entry inhibitors (Maraviroc and AMD3100) (NIH AIDS Reagent Program, United States) was determined with TZM-bl cells, as previously described with modifications (Siddik et al., 2018). Briefly, TZM-bl cells were pre-incubated with a serial dilution, spanning 100–0.01nM, of Maraviroc or AMD3100 in duplicate in a 96-well plate in the presence of 10 μg/ml of DEAE at 37°C for 1 h, and then exposed to an equal amount of infectious virus (∼200 TCID_50_). Forty-eight hour later, the cells were lysed; and β-galactosidase activity was then measured by using a Galacto-Star Reporter Assay System (Applied Biosystems) and a luminometer (Maeda et al., 2001).

### Genotypic Co-receptor Tropism

Prediction of co-receptor tropism was done by using four widely used co-receptor prediction algorithms: Geno2pheno v2.5,^2^ PhenoSeq,^3^ WebPSSM,^4^ and V3 net charge (Delgado et al., 2012; Montagna et al., 2014), according to the procedures described on these websites.

### Statistical Analysis

Statistical analyses were performed with Graph Pad Prism v.6.0b (Graph Pad Software, La Jolla, CA, United States). The non-parametric Kruskal-Wallis test was applied to compare median age, plasma viral loads, and CD4^+^ cell counts between groups. To compare the sex of participants and duration of infection, chi-square (χ^2^) was employed. *A P* value of <0.05 was considered significant.

### Nucleotide Sequence Accession Numbers

The GenBank accession number for sequences are MZ147102–MZ147194.

## Results

### Envelope Subtypes Circulating in Tanzania

A total of 93 full-length infectious envelope sequences were isolated from the plasma viral RNA of 52 treatment-naïve, HIV-1-infected patients in Dar es Salaam, Tanzania. The subtype analysis based on the full-length envelope sequences showed that the most abundant ones were identified as the isRFs (34.6%), followed by subtype A1 (28.8%), C (23.1%), and D (13.5%) (Table 1), indicating co-circulating multiple non-B subtypes and isRFs in this region and in good agreement with previous studies (Hoelscher et al., 2001; Vasan et al., 2006; Shao et al., 2014; Billings et al., 2017; Barabona et al., 2019). Testing of the duration of infection by a limiting antigen avidity enzyme immunoassay revealed that 82.7 and 17.3% of the participants were considered to be chronically and recently infected, respectively (Table 1). There were modest but statistically significant differences in the prevalence of females and recent infection cases among infecting subtypes, but not in median age, plasma viral load or CD4 count (Table 1).

### Phenotypic Co-receptor Tropism

**FIGURE 1:**
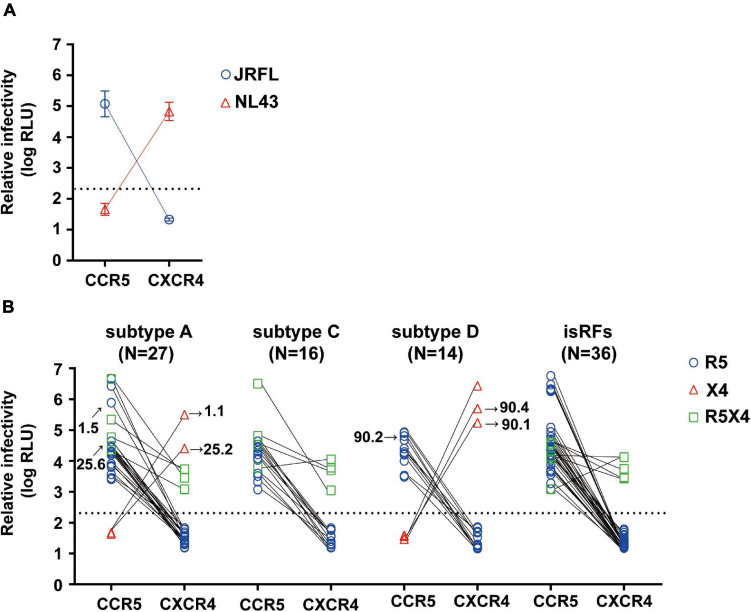
Phenotypic co-receptor tropisms of HIV-1 non-B subtypes isolated in Tanzania. Relative infectivity of lentivirus reporters pseudotyped with control envelopes, NL43 and JRFL **(A)** and a panel of patient-derived envelope clones **(B)** are shown. Target cells were U87.CD4 cells expressing either R5 or X4 co-receptor. Data represent the mean of triplicate assays. The background level of luminescence signal was 200 (2.3log) RLU and is represented by the dotted lines.

We then determined the phenotypic co-receptor utilization of envelope clones as assessed by the pseudovirus infectivity toward U87.CD4^+^ cells expressing either CCR5 or CXCR4 co-receptors as target cells. Pseudoviruses made with the envelopes of JRFL and NL43 established infection exclusively on R5 and X4-expressing U87.CD4^+^ cells, respectively, confirming the validity of this phenotypic co-receptor tropism assay (Figure 1A). Of all 93 pseudoviruses made with patient-derived envelope clones, 80.6% (*n* = 75) and 5.4% (*n* = 5) clones established infection exclusively on R5 and X4-expressing U87.CD4^+^ cells, respectively. Fourteen percent (*n* = 13) of the clones could infect both target cells and were defined as R5X4-tropic (Figure 1B). We observed no correlation between phenotypic tropisms and the duration of infection. Stratification of co-receptor tropism by the subtypes revealed that X4-tropic clones were identified only in subtypes A1 and D; whereas there was no X4-tropic one in subtype C or isRFs (Figure 1B). Both R5 and X4-tropic envelope clones were isolated from 3 subjects, NV01, NV25, and NV90. All clones isolated from patient NV01 (i.e., 1.1 and 1.5) and NV25 (i.e., 25.2 and 25.6) were identified as subtype A1 (Figure 1B), and the genetic distance of intra-patient clones for entire envelope sequences was 3.2 and 3.3%, respectively. In patient NV90, 3 subtype D clones were isolated including 2 phenotypic X4-tropic clones (i.e., 90.1 and 90.4) with the genetic distance of 6.2% and one phenotypic R5-tropic clones (i.e., 90.2). The mean genetic distance for entire envelope sequences between the phenotypic X4-tropic clones and R5-tropic clone was 10.3%.

### Concordance Between Phenotypic Assay and Genotypic Prediction Algorithms

**FIGURE 2:**
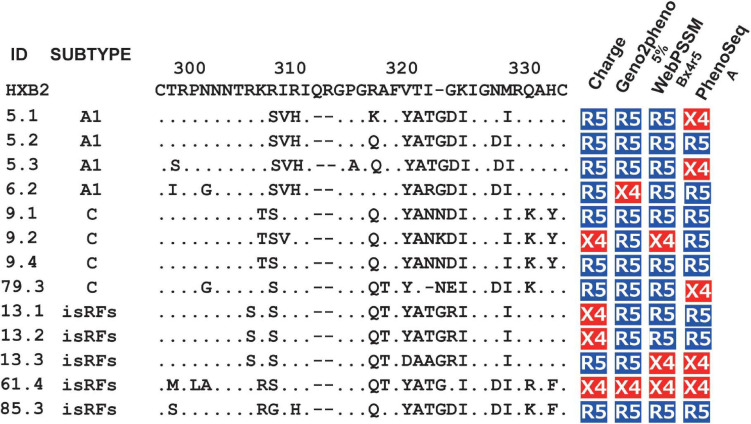
Genotypic prediction of the phenotypic R5X4 clones. Amino acid sequences of V3 region and co-receptor assignment by genotypic analysis of the phenotypic R5X4 clones (*n* = 13). Coreceptor tropism was predicted by V3 net charge, Geno2Pheno [5%], WebPSSM [Bx4r5], and PhenoSeq [A]. The resultant genotype results are shown as R5 (in blue) and X4 (in red).

Next, we tested the four widely used genotypic prediction algorithms, i.e., V3 net charge, Geno2pheno, WebPSSM and PhenoSeq, to infer co-receptor utilization of the clones. For Geno2pheno, we used predetermined false-positive rates (FPRs) of 5 and 10% as widely recommended by several clinical guidelines (Vandekerckhove et al., 2011). We defined the concordance rate as the rate of correct prediction by genotypic prediction algorithms with respect to the phenotypic tropism assay results, and the discordance rate as when the genotypic prediction algorithm contrasted with the phenotypic tropism assay results. For example, when phenotypic assay determines a clone to be R5-tropic, the algorithm will be considered concordant when it predicts the clone to be R5-tropic or discordant when the same clone is predicted to be X4-tropic. We excluded phenotypic R5X4 clones in testing the concordance and discordance of the genotypic prediction. This was because the genotypic analysis of the phenotypic R5X4 clones (*n* = 13; Figure 1B) revealed that one and four clones were predicted to be X4 and R5, respectively, by all algorithms tested, and that the remaining eight clones were differently predicted depending on the algorithms (Figure 2).

**FIGURE 3:**
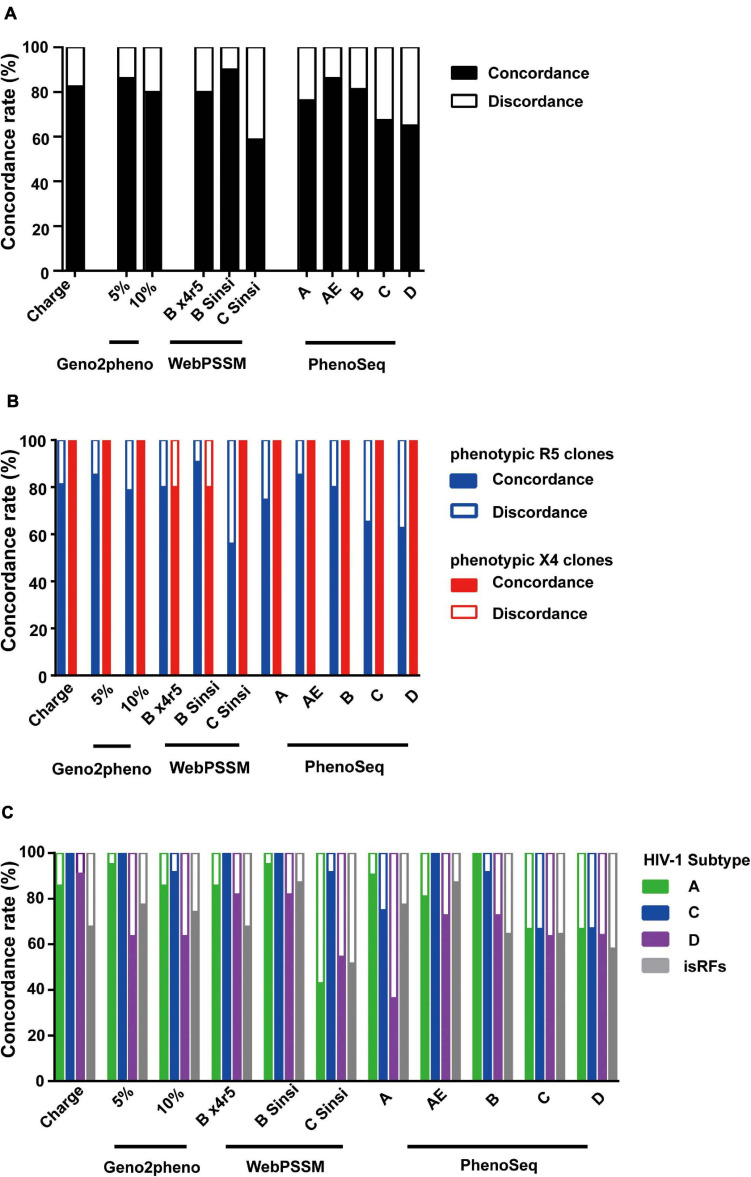
Comparison of phenotypic and genotypic tropism testing. Testing genotypic algorithms of the panel of envelope clones with respect to phenotypic tropism data. The genotypic result was considered concordant when it correctly matched the phenotypic result. **(A–C)** Concordance rate when all clones were shown **(A)**, when phenotypic R5 and X4 clones were separately shown **(B)**, and when phenotypic R5 clones were shown stratified by the pre-determined subtype information **(C)**.

Overall, the mean concordance rate of the genotypic prediction algorithms of V3 net charge (82.5%) and Geno2pheno (83.1%) (Figure 3A) were relatively better than those of WebPSSM (76.3%) and PhenoSeq (75.3%) toward the envelope clones currently circulating in Tanzania. However, within WebPSSM, WebPSSM [B Sinsi], which trained for subtype B syncytium-forming clones, had the highest concordance rate, 90%; whereas WebPSSM [C Sinsi] had the least concordance rate, 58.8% (Figure 3A), suggesting the importance of a trained dataset in the prediction algorithms.

We next analyzed performance of the prediction algorithms within phenotypic R5 and X4 clones. All algorithms predicted better in phenotypic X4 clones with mean concordance rate of 96.4%; whereas in the phenotypic R5 clones, mean concordance rate was only 76.4% (Figure 3B), suggesting the bias to overestimate X4 prediction across all algorithms tested.

We then stratified the clones to viral subtypes, and asked whether performance of the genotypic prediction algorithms would be influenced by the subtypes and isRFs. Across all prediction algorithms tested, clones belonging to subtype D and isRFs showed lower concordance rates of 67.8 and 70.7%, respectively, compared to those of subtype A1 (89.5%) and C (88.7%) (Figure 3C). The results suggested the limitation of tested algorithms in predicting genotypic tropisms of subtype D and isRFs. Of note, the concordance rate of WebPSSM [C Sinsi] increased to 91.7% when predicting only subtype C sequences (Figure 3C), although this algorithm had the least concordance rate of 58.8% when predicting all sequences (Figure 3A).

### Genotypic Prediction of Phenotypic R5-Tropic Clones

**FIGURE 4:**
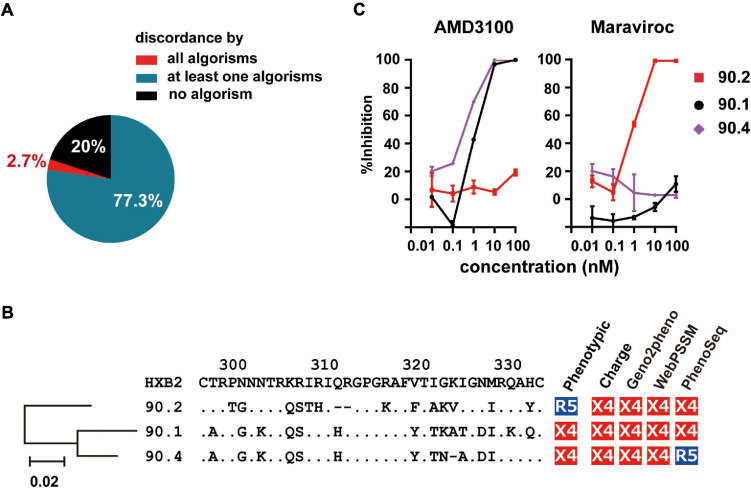
Frequency of incorrect prediction among R5 envelope clones. **(A)** Pie chart showing the frequency of the clones correctly or incorrectly predicted by the genotypic algorithms. **(B)** Phylogenetic tree, amino acid sequences of V3 region, and co-receptor assignment by phenotypic and genotypic analysis of the indicated clones from patient NV90. **(C)** Sensitivity of lentivirus reporters pseudotyped with the indicated envelope clones toward the entry inhibitors, Maraviroc and AMD3100. Target cells were TZM-bl cells. The data represent the mean of triplicate assays.

Next, we scored the concordance of the genetic algorithms in each phenotypic R5-tropic envelope clone (*n* = 75). Only 20% (*n* = 15) of them were correctly predicted by all algorithms tested (Figure 4A); whereas 2.7% (*n* = 2), which belonged to subtype D and isRFs, were discordantly predicted by any algorithms tested. Because one of these cases, clone 90.2, and the other 2 (90.1 and 90.4) were isolated from the same individual, NV90 (Figure 1B), we analyzed the phylogeny of these 3 clones as well as their amino acid sequences at the V3 region (Figure 4B). V3 sequences of all clones harbored relatively many basic amino acids, resulting in an increased net positive charge in the V3 region (>+5), a characteristics typical of X4-tropic tropism. In addition, a potential *N*-linked glycosylation site was found in the phenotypic R5-tropic clone, 90.2 at position 301–303, but not in the phenotypic X4 clones, 90.1 and 90.4 (Figure 4B), suggesting the presence and absence of *N*-linked glycosylation may affect phenotypic tropisms of these clones as previously described in envelope clones in subtype B and CRF01_ AE (Ogert et al., 2001; Tsuchiya et al., 2013; Li et al., 2019). The entry inhibitor sensitivity assay revealed that clone 90.2 was clearly sensitive to Maraviroc and insensitive to AMD-3100; whereas the 2 other clones, 90.1 and 90.4, were *vice versa* (Figure 4C), corroborating the phenotypic assay results with U87 cells.

## Discussion

In this study, we investigated the phenotypic co-receptor usage of 93 envelope clones isolated from the plasma viral RNA of 52 treatment-naïve HIV-infected patients in Tanzania, where multiple non-B viral subtypes and isRFs actively co-circulate. Our findings revealed that the majority of clones (80.6%) phenotypically utilized the R5 co-receptor for entry, with only 5.4% clones having done so using the X4 co-receptor. These latter clones were observed in subtypes A1 and D, but not in C or isRFs. This finding is in line with previous studies describing subtype-specific preference in utilizing the co-receptor for entry (Tscherning et al., 1998; Zhang et al., 2009; Schuitemaker et al., 2010). However, we found that 25% of subtype C clones utilized both R5 and X4 co-receptors for entry; although the previous literature, based on studies that were done >10 year ago in South Africa and Botswana (where subtype C dominated the epidemic) reported that the majority of subtype C clones utilized R5 (Ndung’u et al., 2006; Lin et al., 2011). Thus, the results in this study highlight increasing genetic and phenotypic variability of viral strains in a region where multiple non-B subtypes and isRFs co-circulate, a hallmark of the HIV-1 epidemic in East Africa (Amogne et al., 2016; Siddik et al., 2018).

Genotypic prediction algorithms, including V3 net charge, Geno2pheno, and WebPSSM used in this study, were developed as alternatives to complex phenotypic assays to infer co-receptor tropisms by using subtype B training data set in developed countries where subtype B was dominated (Jensen et al., 2003; Lengauer et al., 2007; Montagna et al., 2014). Previously published literature reported limitation of these algorithms to infer co-receptor tropisms of non-B subtypes and pointed out the overestimation of X4-tropism by them when used in regions where non-B subtypes dominated (Mulinge et al., 2013; Siddik et al., 2018). In line with those findings, the results reported here using clonal envelope sequences demonstrated that the prediction accuracy was different among subtypes; and in particular, we noticed lower prediction accuracy among subtype D and isRFs, which was likely due to limited availability of trained datasets for these viral strains. Alternatively, envelope sequences of this group may have unique characteristics that may differentially affect phenotypic tropisms. The data demonstrating the phenotypic tropisms of clonal non-B subtype sequences here could be helpful to improve prediction accuracy in further studies.

It is reported that patients infected with subtype D show a faster progression of disease than those with subtype A, based on a longitudinal cohort analysis in Tanzania and other sub-Saharan countries with co-circulation of multiple subtypes (Vasan et al., 2006; Amornkul et al., 2013; Venner et al., 2016). However, we did not find any significant association between co-circulating subtypes and isRFs with disease progression as represented by CD4 count or plasma viral load. This lack might have been due to an insufficient number of test subjects in this study or, alternatively, to a tendency toward an increase in emerging isRF cases, which may compromise the analysis of subtype-specific disease phenotypes.

Some limitation of this study merit mention. Although we analyzed phenotypic and genotypic co-receptor tropisms of 93 envelope clones isolated from plasma viral RNA of 52 treatment-naïve, HIV-1-infected patients, this panel did not capture the entirety of envelope genetic diversity in viruses circulating in Tanzania and East Africa. Also, this panel did not capture minor variants within patients that might have different co-receptor tropisms. Indeed, in the present study, we demonstrated the link between clonal sequence diversity and differential phenotypic tropisms in a patient with subtype D infection. Our study demonstrated a snap shot of co-receptor tropisms of viral variants currently circulating in an epidemic region in Tanzania. However, due to the nature of the cross-sectional setting of this study, time-course changes in co-receptor tropisms in a given patient could not be assessed. Despite these limitations, our study provides evidence that viral co-receptor tropism and its prediction of individual envelope clones are influenced by the co-circulation of multiple non-B subtypes and emerging isRFs. The dataset presented here provide a valuable opportunity to train and validate prediction algorithms toward left-behind HIV-1 envelopes belonging to non-B subtypes and isRFs.

## Data Availability Statement

The HIV-1 envelope sequence data presented in the study are deposited in the GenBank accession number MZ147102–MZ147194.

## Ethics Statement

The studies involving human participants were reviewed and approved by Senate Research and Publications Committee of Muhimbili University of Health and Allied Sciences National Health Research Ethics Committee of the National Institute of Medical Research, Tanzania. The patients/participants provided their written informed consent to participate in this study.

## Author Contributions

GPJ and TU: conceptualization. GB, DK, MM, TST, SO, ASM, TK, SM, BS, EL, and KT: reagents and specimens. GPJ: data collection. GPJ, GB, DK, MM, TST, KT, and TU: manuscript writing. BS, EL, and TU: supervision. DK, MM, and TU: funding acquisition. All authors contributed to the article and approved the submitted version.

## Conflict of Interest

The authors declare that the research was conducted in the absence of any commercial or financial relationships that could be construed as a potential conflict of interest.

## Footnotes

1 http://dbpartners.stanford.edu:8080/RegaSubtyping/stanford-hiv/typingtool/

2 https://coreceptor.geno2pheno.org/

3 http://tools.burnet.edu.au/phenoseq/

4 https://indra.mullins.microbiology.washington.edu/webpssm/index2.html
